# Real-time digital pathogen surveillance — the time is now

**DOI:** 10.1186/s13059-015-0726-x

**Published:** 2015-07-30

**Authors:** Jennifer Gardy, Nicholas J. Loman, Andrew Rambaut

**Affiliations:** Communicable Disease Prevention and Control Services, British Columbia Centre for Disease Control, 655 West 12th Avenue, Vancouver, BC V5Z 4R4, Canada; Institute of Microbiology and Infection, University of Birmingham, Birmingham, B15 2TT UK; Institute of Evolutionary Biology, University of Edinburgh, Edinburgh, EH9 2FL UK

**Keywords:** Ebola, Real-time whole-genome sequencing, Surveillance, Salmonella

## Abstract

It is time to shake up public health surveillance. New technologies for sequencing, aided by friction-free approaches to data sharing, could have an impact on public health efforts.

## Background

Zoonotic pathogens have serious consequences for human health. A single transmission of a virus — plausibly from a bat to a boy playing by an old, dead tree [1] — has led to the largest outbreak of Ebola virus disease ever, claiming over 11,000 lives in West Africa [2]. Similarly, the MERS coronavirus, seemingly endemic in dromedary camels in the Arabian Peninsula [3], has repeatedly spilled over to humans, causing numerous outbreaks — the latest involves hundreds of patients in South Korea and China and arose from a single infected traveller. In 2009, a new lineage of swine H1N1 influenza emerged in North America into humans, creating the first pandemic of the 21st century and establishing a new seasonal lineage of influenza A virus. Over a million cases of *Salmonella* infection occur in the USA each year [4], and, when outbreaks are large enough to warrant investigation, they are often linked to sources of food production.

## The need for pathogen genomic surveillance

Controlling potentially lethal pathogens requires timely, comprehensive surveillance systems. These systems currently rely on case counting and simple genotyping techniques, but surveillance could be markedly improved through genomics. Pathogen sequencing has commonly been used for the identification of isolates and their classification into ‘genotypes’ (genetic lineages) that can be associated with host species or geographical regions. More recently, however, genome sequencing has emerged as a crucial tool in our real-time response to outbreaks of infectious disease.

Many pathogens causing acute disease evolve rapidly, with genomes from even closely linked cases often exhibiting discernable nucleotide differences over time-scales of weeks to months — differences that can be used to draw informative epidemiological conclusions. Initial genetic sequencing of a newly emerging virus can provide a substantial amount of information about the nature of the pathogen through comparison with existing sequences. As more cases are sequenced, analysis of the genetic diversity among the population can provide estimates of how fast the disease is spreading and help predict its future course. In the early stages of the 2009 H1N1 influenza A pandemic, a sequence-based estimate of the transmission potential was shown to be comparable to a traditional epidemiological estimate, providing the first characterization of the epidemiology of the new human epidemic [5]. Sequencing of bacterial pathogens has also proved useful in understanding and managing acute outbreaks in hospital and community settings.

## Real-time genomic epidemiology: a new opportunity

Such progress is remarkable, but we are now on the cusp of a second revolution in genomic epidemiology. The potential exists to move from pathogen genomics providing static ‘snapshots’ of epidemics, often months after the cases occurred, to a situation where data are produced in real-time, providing a detailed picture of the epidemic that is only a few days old. The advance of high-throughput sequencing means that it is possible to obtain whole-genome sequences from clinical samples within days. Now, genomic epidemiologists are working to bring sequencing to the outbreak, rather than sending isolates to a reference laboratory. Such rapid results are crucial if the intention is to intervene in an outbreak rather than simply document it in retrospect. For such applications, the useful half-life of genomic information for epidemiology, while there is a chance to influence control practices, can be measured in days or weeks.

Benchtop sequencing technologies and, most recently, portable nanopore sequencing are making cheap, close-to-the-sample sequencing a reality. One of us (NJL) recently showed that the Oxford Nanopore MinION can be used for real-time detection of *Salmonella enterica* from clinical isolates during the investigation of a large hospital outbreak [6]. Others have used this system for metagenomics diagnosis of viral infections directly from clinical samples [7].

## Sharing and comparing

No matter how rapidly they are generated, pathogen genome sequences are of limited utility when viewed in isolation. They must be examined in the context of a constantly updating database of comparator strains and the associated epidemiological and surveillance data. This can be seen in the *S. enterica* hospital outbreak. Initially, genome sequencing demonstrated that the outbreak was of a single strain, but could not identify its origins. When the genome data were integrated with national surveillance data, the cases were linked to a larger, national outbreak. Later, when the UK data were compared with European sequences, a link to a larger, continental outbreak became apparent and was traced to an egg production factory [2]. Sharing in this case was ad hoc — sequences generated during the outbreak were shared through the file-sharing utility Dropbox (http://www.dropbox.com) with Public Health England, who were then able to integrate the genomic data with their national surveillance database. Indeed, the modern internet sharing era is ideally suited for such digital pathogen surveillance data. Cloud services, such as Google Drive (http://drive.google.com) and Dropbox, facilitate rapid data sharing between engaged parties, while collaborative tools, such as GitHub (http://www.github.com) and the Slack communication platform (http://www.slackhq.com), make it easy for international teams to write to an ‘open lab notebook’ [8]. Online tools can provide powerful evolutionary and epidemiological visualizations, such as Bedford and Nehrer’s nextflu, which was originally designed for influenza strain tracking and has now been adapted to integrate real-time Ebola genomic datasets (http://ebola.nextflu.org) [9]. We have also employed the Microreact website (http://microreact.org) to integrate phylogenetic and geographical information into an easy-to-use website for epidemiologists engaged in the Ebola outbreak response [10].

## Into the field

Real-time genomic surveillance can also be performed in resource-limited settings. Since April 2015, we have been engaged in genomic surveillance of the ongoing outbreak of Ebola virus in West Africa. Under the auspices of the European Mobile Laboratories in Guinea, we have successfully used nanopore sequencing to cluster cases based on their genome sequence. These clusters are available within a few days after diagnosis and can be used by epidemiologists to investigate chains of transmission. Another group, based at the University of Cambridge, UK, have been providing similar information generated on the Ion Torrent platform in real-time from Sierra Leone. Integration of the most-recent datasets showed that contemporary cases in Guinea cluster with cases in Sierra Leone, strongly suggestive of cross-border transmissions [11].

Such ad hoc international collaborations seem to arise naturally in the context of urgent public health investigations, where there is strong pressure to release data and a community of researchers ready to engage (see also the *Escherichia coli* O104:H4 outbreak in Germany, where crowd-sourced analysis was carried out by researchers on four continents) [12]. However, challenges exist in standardizing and scaling this model for routine pathogen surveillance. Such activities are typically performed under the purview of government-funded surveillance laboratories. Although there are good arguments that agencies should share their data widely and openly, there are concerns about negative side effects. Many objections center around how potentially identifying ‘metadata’ is shared whilst protecting anonymity of individuals to avoid possible breaches of privacy. Institutions or companies, for example those identified by genomics as the source of an outbreak, could suffer financial losses or reputational damage. Clearly, a careful balance needs to be reached between these concerns and the evident positive public health benefit of routine and rapid data sharing.

## Concluding remarks

We envisage a future of ubiquitous pathogen surveillance, generated with ‘cheap as chips’ sequencing, linked by the internet. Automated clustering algorithms will be able to detect outbreaks far quicker than traditional case-exceedance methods [13]. Portable genome sequencing will make it possible to sample the natural environment for pathogen reservoirs, as well as clinical samples close to the patient. It is our firm belief that these digital genomic data, generated by a diverse range of agents — and then shared widely — will serve to break conventional silo thinking about infections.
